# Comparative RNA-Seq profiling of berry development between table grape ‘Kyoho’ and its early-ripening mutant ’Fengzao’

**DOI:** 10.1186/s12864-016-3051-1

**Published:** 2016-10-12

**Authors:** Da-Long Guo, Fei-Fei Xi, Yi-He Yu, Xiao-Yu Zhang, Guo-Hai Zhang, Gan-Yuan Zhong

**Affiliations:** 1 College of Forestry, Henan University of Science and Technology, Luoyang, 471003 Henan Province China; 2 USDA-Agricultural Research Service, Grape Genetics Research Unit, Geneva, NY 14456 USA

**Keywords:** Grape, Early ripening, RNA-Seq, Bud mutant, Kyoho

## Abstract

**Background:**

Early ripening is an important desirable attribute for fruit crops. ‘Kyoho’ is a popular table grape cultivar in many Asian countries. ‘Fengzao’ is a bud mutant of ‘Kyoho’ and ripens nearly 30 days earlier than ‘Kyoho’. To identify genes controlling early fruit development and ripening in ‘Fengzao’, RNA-Seq profiles of the two cultivars were compared at 8 different berry developmental stages in both berry peel and flesh tissues.

**Methods:**

RNA-Seq profiling of berry development between ‘Kyoho’ and ‘Fenzhao’ were obtained using the Illumina HiSeq system and analyzed using various statistical methods. Expression patterns of several selected genes were validated using qRT-PCR.

**Results:**

About 447 millions of RNA-Seq sequences were generated from 40 RNA libraries covering various different berry developmental stages of ‘Fengzao’ and ‘Kyoho’. These sequences were mapped to 23,178 and 22,982 genes in the flesh and peel tissues, respectively. While most genes in ‘Fengzao’ and ‘Kyoho’ shared similar expression patterns over different berry developmental stages, there were many genes whose expression were detected only in ‘Fengzao’ or ‘Kyoho’. We observed 10 genes in flesh tissue and 22 genes in peel tissue were differentially expressed at FDR ≤ 0.05 when the mean expression of ‘Fengzao’ and ‘Kyoho’ were compared. The most noticeable one was VIT_214s0030g00950 (a superoxide dismutase gene). This ROS related gene showed lower expression levels in ‘Fengzao’ than ‘Kyoho’ in both peel and flesh tissues across various berry developmental stages with the only exception at véraison. VIT_200s0238g00060 (TMV resistance protein n-like) and VIT_213s0067g01100 (disease resistance protein at3g14460-like) were the two other noticeable genes which were found differentially expressed between the two cultivars in both peel and flesh tissues. GO functional category and KEGG enrichment analysis of DEGs indicated that gene activities related to stress and ROS were altered between the two cultivars in both flesh and peel tissues. Several differentially expressed genes of interest were successfully validated using qRT-PCR.

**Conclusions:**

Comparative profiling analysis revealed a few dozens of genes which were differentially expressed in the developing berries of ‘Kyoho’ and its early ripening mutant ‘Fengzao’. Further analysis of these differentially expressed genes suggested that gene activities related to ROS and pathogenesis were likely involved in contributing to the early ripening in ‘Fengzao’.

**Electronic supplementary material:**

The online version of this article (doi:10.1186/s12864-016-3051-1) contains supplementary material, which is available to authorized users.

## Background

Fruit ripening is a complex and highly coordinated developmental process. A series of changes in physiological and biochemical catabolism are involved during fruit ripening, which in turn affect fruit quality, such as flavor, texture, color and aroma [1]. Fleshy fruits have been divided into climacteric and non-climacteric categories. Grapes are non-climacteric fruit and their berry development follows a double sigmoid pattern, involving two phases of rapid growth separated by a lag phase [2]. The growth and developmental stages of grape berry have been well characterized and described in the modified E-L system [2]. Recently, a number of transcriptional and metabolomic analyses have been carried out to study the grape berry ripening process at various berry developmental stages, among different cultivars, and in distinct environmental conditions [3–6]. These studies have uncovered a wealth of developmentally regulated genes in grape berry [7, 8]. However, much remains to be understood about the molecular and biochemical events leading to grape ripening [7].

Most of our knowledge about fruit development and ripening mechanisms come from climacteric fruits [1, 7], especially from the characterization of monogenic tomato mutants, including ripening inhibitor (*rin*), non-ripening (*nor*), colorless non-ripening(*Cnr*), green-ripe (*Gr*), green flesh (*gf*), high pigment 1 (*hp1*), high pigment 2 (*hp2*), and never-ripe (*Nr*) [1]. In perennial fruit species, it is not very easy to generate and screen such ripening mutants as what have been done in tomato. Nevertheless, mutants affecting fruit development have been reported in several fruit species, including pear [9] and sweet orange [10]. Some studies have been attempted to understand the molecular and biochemical processes of wild types and their mutants in these perennial fruit species. For example, based on a combination of two-dimensional electrophoresis (2-DE) and matrix assisted laser desorption ionization-time of flight mass spectrometry (MALDI-TOF MS) analysis, Liu et al. [9] detected an increase of proteins related to cell-wall modification, oxidative stress and pentose phosphate metabolism and a decrease of proteins related to photosynthesis and glycolysis during the fruit development process in both ‘Zaosu’ pear and its early-ripening bud sport. However, all these proteins increased or decreased much faster in the early-ripening bud sport than its wild type ‘Zaosu’. Another example is the comparative profiling study of the late-ripening orange mutant of ‘Jincheng’ (*C. sinensis* L. Osbeck) and its wild type, discovering that differentially expressed unigenes (DEGs) were mainly clustered into five pathways: metabolic pathways, plant-pathogen interaction, spliceosome, biosynthesis of plant hormones and biosynthesis of phenylpropanoids [10]. From these and other work, many genes and pathways have clearly been demonstrated to involve in the fruit ripening processes of perennial species.

‘Kyoho’is a tetraploid interspecific hybrid grape derived from a cross of *Vitis vinifera* x *Vitis labrusca*. It is a well-known table grape cultivar widely grown in China, Japan and several other Asian countries. It has large berries and high yield, and can grow well in high temperature, rainy, wet and other adverse environments. However, ‘Kyoho’ is a mid-late ripening grape, which limits it availability for consumers in the early seasons. Recently, we identified an early ripening bud mutation of ‘Kyoho’ and released it as an early table grape, named as ‘Fengzao’ [11]. ‘Fengzao’ ripens in early July in Henan province, China, nearly one month earlier than ‘Kyoho’, while there are no noticeable changes of other fruit and horticultural traits between the two cultivars [12]. As a step to understand the genetic basis of early ripening in ‘Fengzao’, we compared the RNA-Seq profiles of “Kyoho’ and ‘Fengzao’ at 8 different berry developmental stages in both berry peel and flesh tissues. We identified several genes which likely play critical roles in accelerating the berry ripening process in ‘Fengzao’.

## Materials and methods

### Plant material and RNA preparation

**Fig. 1:**
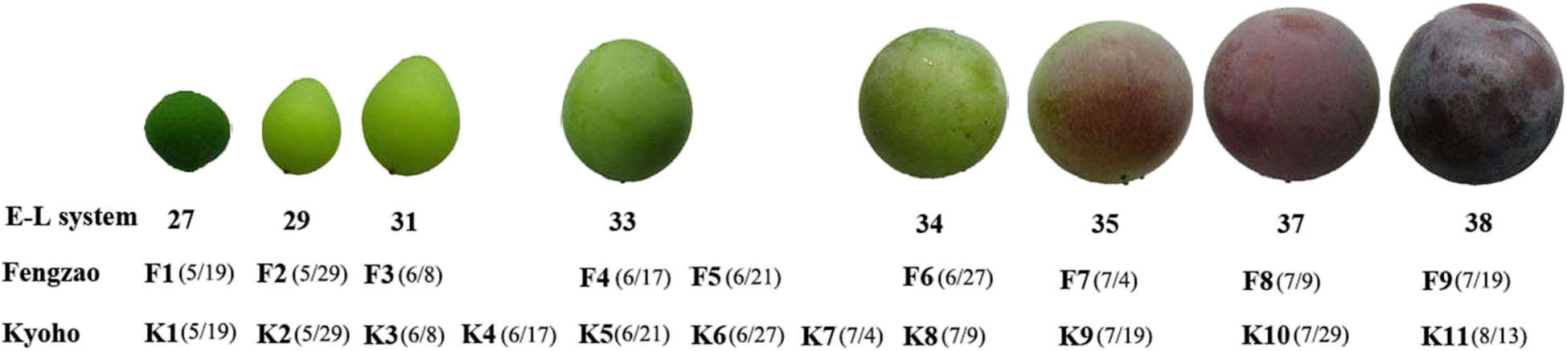
Berry developmental stages and sampling time points. The E-L system follows that of Coombe [2]. F1-F9 and K1-K11 represent the sampling time points for MT and WT, respectively. Because WT and MT had different ripening dates, the corresponding dates for reaching their E-L system stages, as indicated in the brackets after F or K, were different. For example, F4 and K5 both represented the samples taken at the E-L system stage 33, but K5 was sampled later (6/21) than F4 (6/17). This is because ‘Fengzao’ reached the E-L system stage 33 about 5 days earlier than ‘Kyoho’

Both ‘Kyoho’ (hereafter WT) and ‘Fengzao’ (hereafter MT, clone number: 200203 F9) vines were cultivated in the same vineyard with the same viticulture management practices on the farm of Henan University of Science & Technology (the county of Yanshi, Luoyang, China (34.41° N, 112.46° E)). The mean annual temperature is 14.2 °C. During the period of early April and late September, the average day length is 13.8 h. Fruit samples from three vines in 2013 were harvested at the developmental stages corresponding to EL 27, 29, 31, 33, 34, 35, 37, and 38 (EL refers to the modified Eichhorn and Lorenz developmental scale as described by Coombe [2]). The characteristics of these developmental stages are as follows: EL 27 at the beginning of berry setting; EL 31 pea-size berries; EL 32 beginning of bunch closure, berries touching; EL33 characterized by hard green berries; EL 34 just before véraison characterized by green berries, which are starting to soften; EL35 corresponding to véraison; EL 37 involving sugar and anthocyanins accumulation, and active growth; and EL 38 corresponding to harvest time [2]. We collected berry samples from both MT and WT on the same dates when MT or WT reached its eight berry developmental stages. Because MT rapidly accelerated its ripening process after E-L 31, the time (days) required to reach to the E-L stages 33, 34, 35, 37 and 38 in MT were different from that in WT (Fig. 1). As a result, we have 20 berry bulk samples taken. These 20 bulk samples included 9 (F1-F9) from MT and 11 (K1-K11) from WT. Among the 9 MT berry samples, F1, F2, F3, F4, F6, F7, F8 and F9 each represented one of the 8 E-L berry developmental stages of MT (Fig. 1). F5 did not correspond to any of the 8 E-L stages in MT. It was taken at the same date (6/21) when WT reached its E-L stage 33 and the K5 sample of WT was harvested. Similarly, among the 11 WT berry samples, K1, K2, K3, K5, K8, K9, K10 and K11 each represented one of the 8 E-L berry developmental stages of WT (Fig. 1). The other 3 bulk samples, K4 (6/17), K6 (6/27) and K7 (7/4), were taken when MT reached its E-L stages 33, 34 and 35, and the F4, F6, and F7 samples of MT were harvested, respectively.

The RNA-Seq libraries from the peel and flesh tissues of WT were labeled as KP and KF, respectively. Similarly, the corresponding libraries of MT were labeled as FP and FF. Ten representative berries were sampled at each developmental stage from 3 individual vines in 2013. Flesh and peel tissues were separated and immediately frozen in liquid nitrogen and stored at -80 °C until used for RNA extraction. Representative berries were similarly sampled and processed in 2014 for qRT-PCR validation of the expression levels of certain genes of interest.

### Library preparation and sequencing

RNA-Seq libraries were constructed according to the protocols of Zhong et al. [13] and Wang et al. [14]. Briefly, 20 μg total RNA was used to enrich mRNA by using the oligo (dT) magnetic beads. After adding first strand synthesis buffer, the mRNA was fragmented by incubation at 94 °C for 5 min. The first strand cDNA was synthesized with random hexamer-primer using the fragmented mRNAs as templates by Superscript®III Reverse Transcriptase (Invitrogen, Carlsbad, CA, USA). cDNAs were purified with Agencourt RNAClean XP beads (Beckman Coulter Genomics, Danvers, MA, USA) followed by end repair and dA-tailing (NEB, Ipswich, MA, USA). The short fragments were then ligated with Y-shaped adapters using high concentrated T4 ligase (Enzymatics, Beverly, MA, USA). The adaptor ligated cDNAs were size selected with Ampure XP beads (Beckman Coulter Genomics, Danvers, MA, USA) before PCR amplification with indexed primers. RNA-Seq libraries were sequenced using the Illumina HiSeq system at the Biotechnology Resource Center of Cornell University (Ithaca, NY, USA). In total, forty libraries were sequenced from fruit peel and flesh of WT and MT sampled at different developmental stages. All sequences were deposited in the Short Read Archive at NCBI under accession number SRR1557134 and SRR1558172.

### Sequence and expression analysis

The raw reads were cleaned by removing adaptor sequences, empty reads and reads with unknown or low-quality bases. The clean reads were aligned to the grape reference genome [15] using TopHat v2.0.9 with default parameters. The coordinates of the mapped putative transcripts were then compared with the current grape genome V2.1 annotation [16] (http://genomes.cribi.unipd.it/). The software of featureCounts was used to obtain raw read counts from the alignments which can be unambiguously assigned to genomic features (exon) for each sample [17]. The R package, DESeq2 [18], was employed to identify differentially expressed genes (parameters: p value ≤ 5 %) based on the read count for each gene at different developmental stages.

### Cluster analysis and gene annotation

Following alignments, raw counts of individual genes were normalized to Reads Per Kilobase of exonmodel per Million mapped reads (RPKM) based on the 12X v2.1 gene annotation [16] using featureCounts [17]. To correct potential scale effect of gene expression and avoid working with negative expression values, we added 1 to the RPKM values of the expressed genes and then transformed the modified PRKM values using 2 as the log base. A mean expression value was calculated across different berry developmental stages for each expressed gene. The mean calculation did not include those data points with log2 value being 0. Those data points which had no detectable expression were replaced with the mean for the purpose of pattern comparison. The deviation from the mean expression was calculated for each expressed gene at individual berry developmental stages. The expression change patterns of individual genes were characterized by K-means clustering in both MT and WT. All statistical analyses were performed in R version 2.15.3. Clustering of transcript expression patterns based on RPKM levels was carried out using the k-means method and with the Euclidean similarity metric. Gene Ontology (GO) categorization was carried out using Blast2GO (version 2.3.5) (http://www.blast2go.de/) with the 12X V2.1 predicted transcripts as references [16]. Then, WEGO software (http://wego.genomics.org.cn/cgi-bin/wego/index.pl) was used to perform GO functional classification (biological process, molecular function and cellular component) for all unigenes. Functional analysis and enrichment of biological processes were performed using the BiNGO 2.44 plugin tool in Cytoscape version 3.3.0 with adjusted p-values using the Benjamini & Hochberg correction [19]. Kyoto Encyclopedia of Genes and Genomes (KEGG) pathway enrichment analysis was performed using KOBAS 2.0 (http://kobas.cbi.pku.edu.cn/home.do) [20].

### Real-time Quantitative RT-PCR Validation

Expression of a selected group of differentially expressed genes identified by the RNA-Seq analysis was re-examined through the real-time quantitative RT-PCR (qRT-PCR) analysis. The grape ubiquitin1 gene (GenBank Accession number CA808925) amplified with the primers F (5′- GTGGTATTATTGAGCCATCCTT -3′) and R (5′- AACCTCCAATCCAGTCAT CTAC -3′) was used as internal control. The primer sequences for validation of the selected genes were designed using Primer 3 (Additional file 1). All qRT-PCRs were performed in a CFX96 Real Time PCR Detection System (Bio-Rad), and the reactions were performed in duplicate for each sample with a reaction volume of 10 μl containing 1.0 μM each primer, 1.0 μl of cDNA, 5 μl of SYBR premix Ex *Taq* (TaKaRa Bio Inc.), and 3.5 μl sterile distilled water and at least three biological replicates were evaluated for each gene tested. The PCR programs are the same as Wang et al. [21] The qRT-PCR data were directly analyzed using the CFX Manager software (Bio-Rad). Normalization of qRT-PCR data was achieved by subtracting the Ct values of the internal reference genes from the Ct values of the target genes to obtain ΔCt.

## Results

**Fig. 2:**
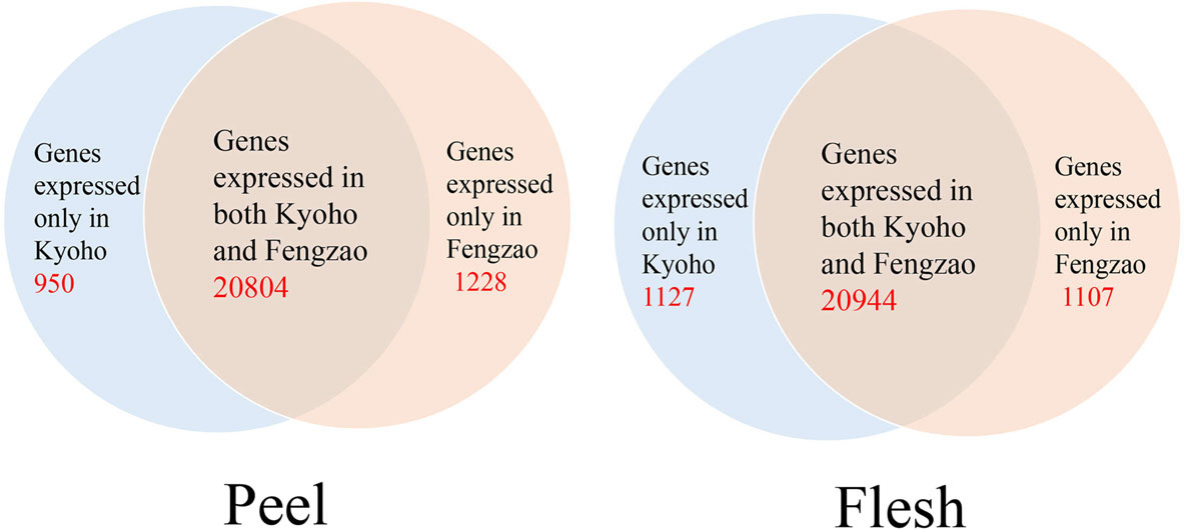
Venn diagrams of uniquely and commonly expressed genes between WT and MT in the flesh and peel tissues

About 11 million RNA-Seq reads of ~100 bp long were obtained for each berry developmental stage for each cultivar. These reads had a Q20 percentage over 95 % (percentage of sequences with sequencing error rate lower than 1 %). In total, about 440 millions of read sequences were successfully obtained from 40 libraries. Of the high quality reads, 62.97 % (Additional file 2) were uniquely mapped to the grape reference genome. Almost 70 % of the uniquely mapped reads were assigned to known exons. On the basis of the reference genome [15, 16], expression profiles of 23,178 and 22,982 genes were generated in this study for the berry flesh and peel tissues, respectively (Fig. 2).

In this study, 31,846 genes are predicted on the basis of the latest grape genome annotation (v2.1 produced by CRIBI; http://genomes.cribi.unipd.it/ grape/) [16]. A similar number of annotated genes were found in both flesh (72.78 %) and peel (72.16 %) tissues. To facilitate global analysis of the gene expression, all predicted genes were assigned to functional categories using Blast2GO (version 2.3.5) (http://www.blast2go. org/). About 69.98 % of the flesh transcriptome and 69.02 % of the peel transcriptome had assigned functions. Pathway-based analysis can help further understand the biological functions and gene interactions. A total of 26,529 annotated sequences were mapped to canonical pathways of KEGG. Overall, the unigenes were assigned to 145 different KEGG pathways.

### Comparison of overall expression patterns between WT and MT

**Fig. 3:**
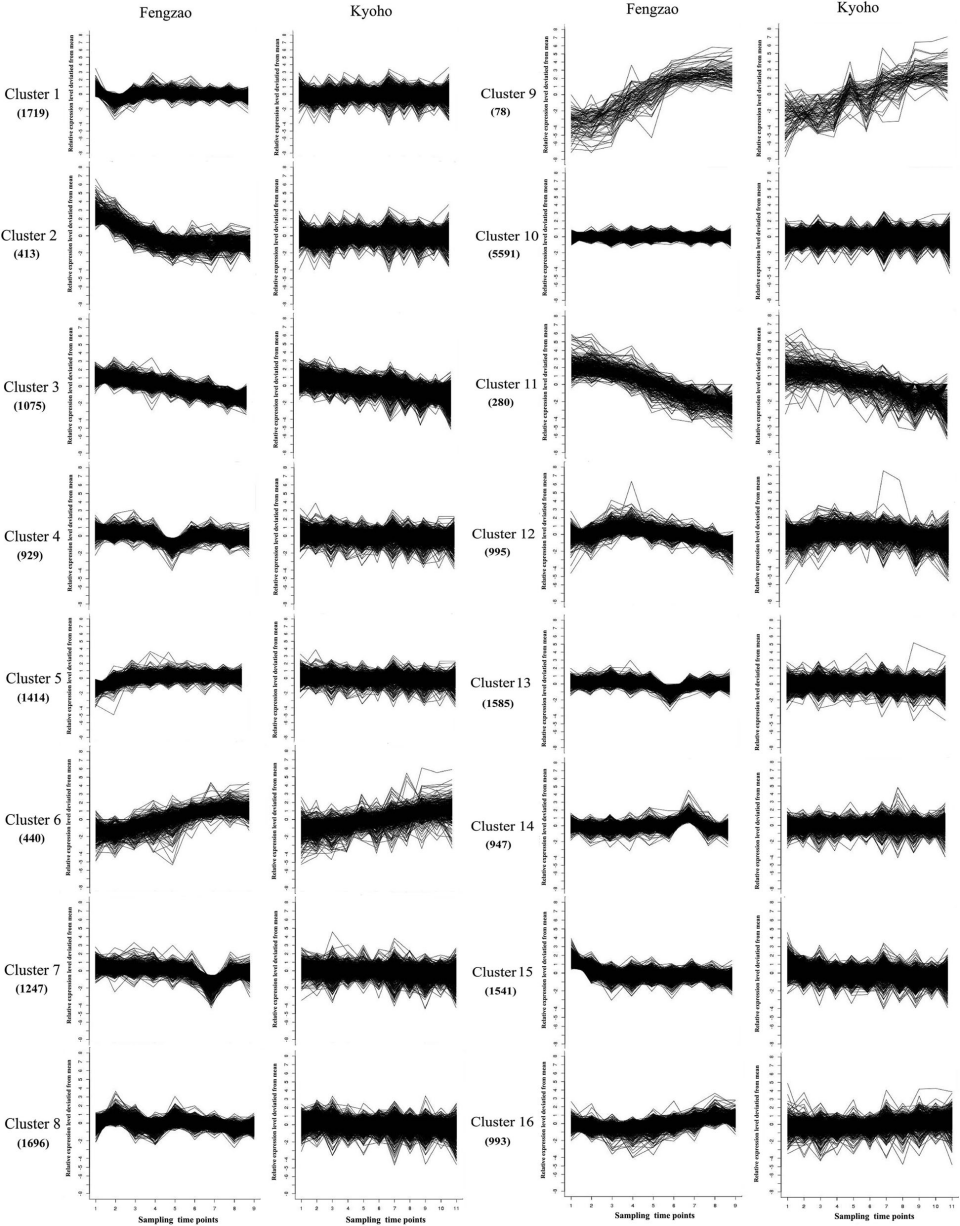
Cluster anlysis of the gene expression patterns in the flesh tissue (20,944) of both WT (Kyoho) and MT (Fengzao) across various developmental stages. Clustering was performed using k-means statistics and the number of genes included in each of the clusters is indicated in the bracket. The Y axis represents deviation of the expression levels (log-transformed, see Methods) of a gene at different developmental stages from the mean expression of the gene in consideration. The X-axis represents the sampling time points

**Fig. 4:**
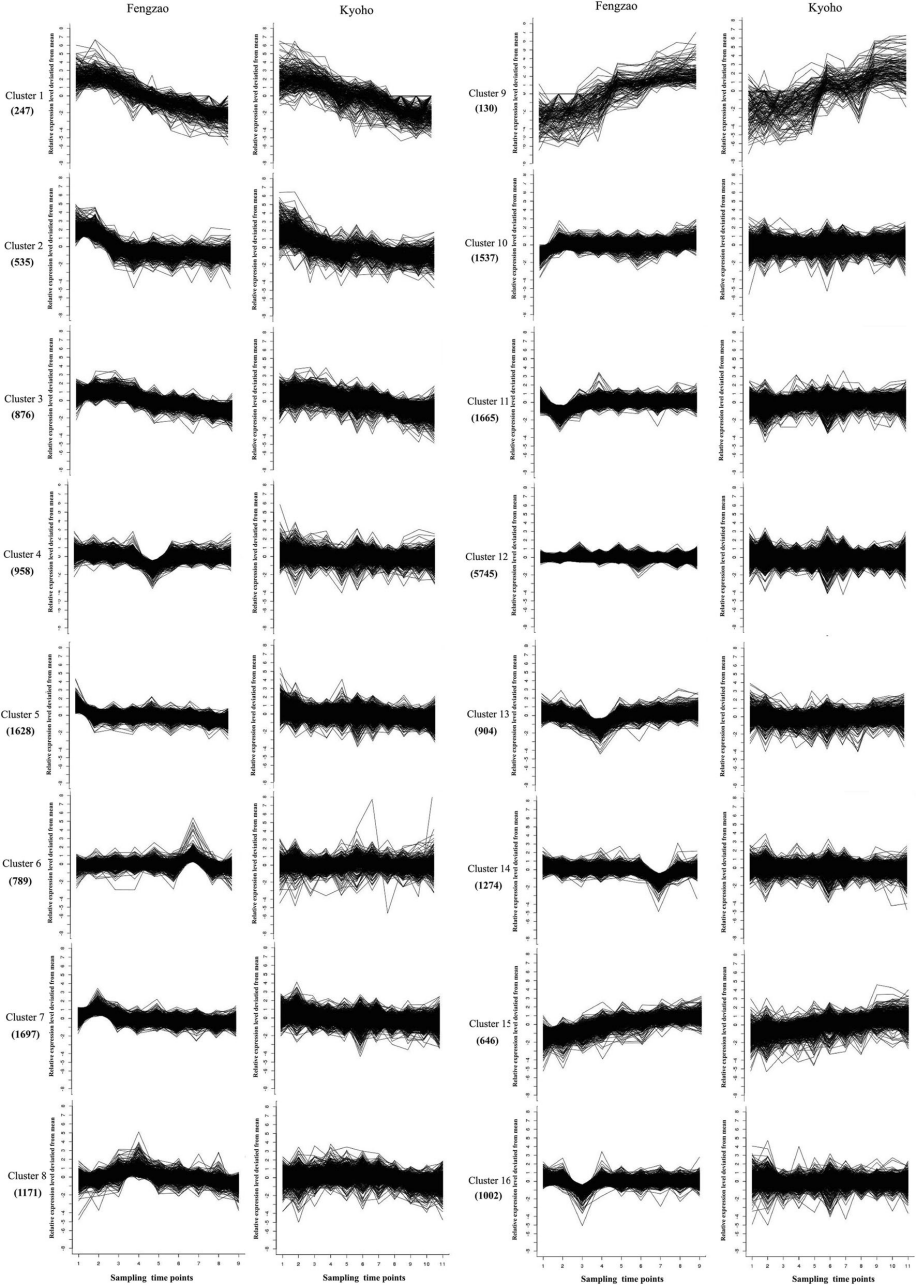
Cluster anlysis of the gene expression patterns in the peel tissue (20,804) of both WT (Kyoho) and MT (Fengzhao) across various developmental stages. Clustering was performed using k-means statistics and the number of genes included in each of the clusters is indicated in the bracket. The Y axis represents deviation of the expression levels (log-transformed, see Methods) of a gene at different developmental stages from the mean expression of the gene in consideration. The X-axis represents the sampling time points

Expression patterns of the genes detected in the flesh (20944) or peel tissue (20844) in both WT and MT were compared using the K-means clustering method. Sixteen groups of WT and MT genes with similar expression patterns were recognized in both peel and flesh tissues (Additional file 3 and Additional file 4). The genes were classified into individual groups in such a way that each pair of groups between WT and MT contained the same set of genes. As a whole, the expression patterns of most cluster groups were very similar between WT and MT (Fig. 3 and Fig. 4). However, there were several interesting exceptions. Firstly, the range of variation within some clusters was much smaller in MT than in WT. Examples include clusters 1, 3, 5, 8, 10 in the flesh tissue and clusters 5, 7, 11, and 12 in the peel tissue. Secondly, genes in some clusters of MT often showed apparent up- or down-regulation of their expression at a particular developmental stage compared with their counterparts in WT. Such examples can be clearly identified in clusters 4, 7, 13, and 14 in the flesh tissue and clusters 4, 6, 13, 14, and 16 in peel tissue. Thirdly, certain clusters of genes showed recognizable patterns of downward or upward expression as berries develop. Clusters 3 and 11 in the flesh tissue and clusters 1 and 3 in the peel tissue were clearly downward while clusters 6 and 9 in the flesh tissue and clusters 9 and 15 were upward. Finally, genes in most clusters, in either flesh or peel tissue, showed more or less consistent expression in both WT and MT. Cluster 2 in MT flesh is a special one in which the expression levels of genes were high at the beginning, and then slowly decreased as berry development advanced. In contrast, expression of the exact same genes didn’t change so much in WT. Most of the genes in this cluster were involved in the pathways of starch and sucrose metabolism (E.C. 3.1.1.11 VIT_200s0323g00050, E.C. 3.2.1.15 VIT_205s0020g00420), galactose metabolism (E.C. 1.1.3.9 VIT_202s0025g02600), and phenylpropanoid biosynthesis (E.C. 4.3.1.25 VIT_206s0004g02620).

### Uniquely expressed genes

**Fig. 5:**
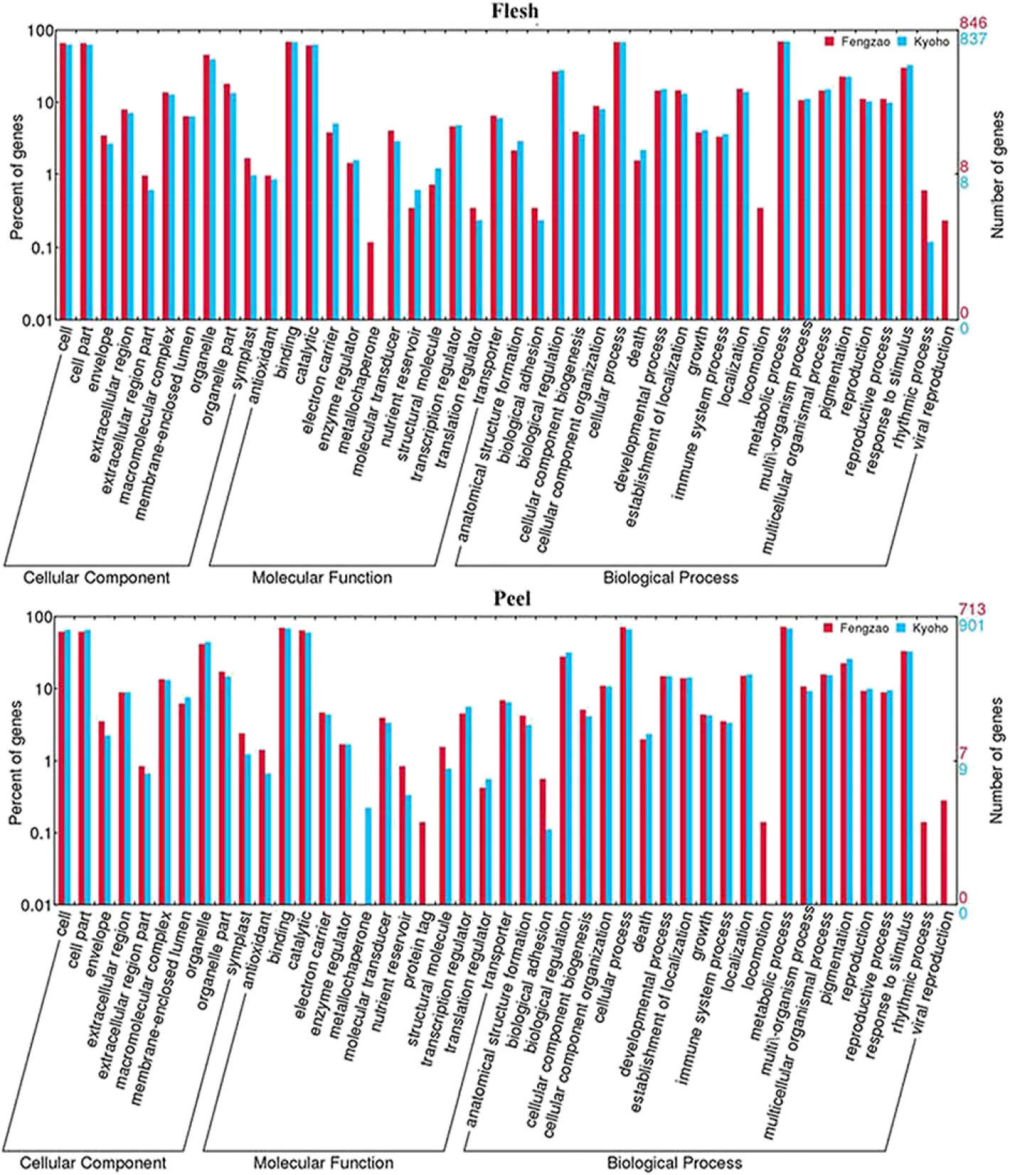
GO analysis of the genes expressed only in the flesh or peel tissue of WT (Kyoho, blue) and MT (Fengzhao, red). The X-axis represents various GO terms in the three main categories of biological processes, cellular components, and molecular functions. The right Y-axis indicates the number of genes in a category. The left Y-axis indicates the percentage of a specific GO term of genes in the corresponding main category

There were many genes which were only detected in WT (1127 in flesh and 950 in peel) or MT (1107 in flesh and 1228 in peel) (Fig. 2). Some of these genes could be falsely identified due to experimental errors, but most of them were likely real. Since MT is a bud mutant of WT, and WT is the base line to compare with, those genes whose expression were detected in MT, but not in WT, were likely up-regulated in MT directly or indirectly by the mutation(s). Similarly, those genes whose expression were detected in WT, but not in MT, were likely down-regulated in MT. Among these “uniquely” expressed genes, only a small number of them could be annotated with known functions. GO analysis of these genes revealed that many different biological processes and molecular functions were represented and shared by both flesh and peel tissue and by both WT and MT (Fig. 5). It was very interesting to note that some processes/functions were only represented in certain tissue or genotype. For example, the biological process of locomotion was represented by some of the genes uniquely expressed in both peel and flesh tissue of MT (Fig. 5), suggesting that expression of these genes involved in the process were up-regulated resulting from the mutation(s) in MT. Rhythmic process was also represented by some of the uniquely expressed genes, but only in the peel tissue of MT. Detailed examination revealed that RR22 (two-component response regulator arr22, VIT_204s0043g00690), ESP (encodes a separase, VIT_213s0106g00110), COL2 (constans-1 CO, VIT_214s0083g00640) and an uncharacterized protein (VIT_205s0165g00005) were involved. These genes are known to be related to the signal transduction and flowering promotion. As for molecular function, protein tag was represented only in the MT peel tissue, while metallo chaperone was in WT peel and MT flesh. VIT_212s0028g00530, for example, is related to the GO term of metallo chaperone. It was only detected in WT peel and MT flesh. Most of the remaining GO terms are similar for the uniquely expressed genes in WT and MT (Additional file 5). The top three GO terms of molecular function detected in both WT and MT are: oxidoreductase activity; tetrapyrrole binding; and heme binding. KEGG enrichment analysis revealed that the most represented were starch and sucrose metabolism pathways.

### Differentially expressed genes between WT and MT

**Fig. 6:**
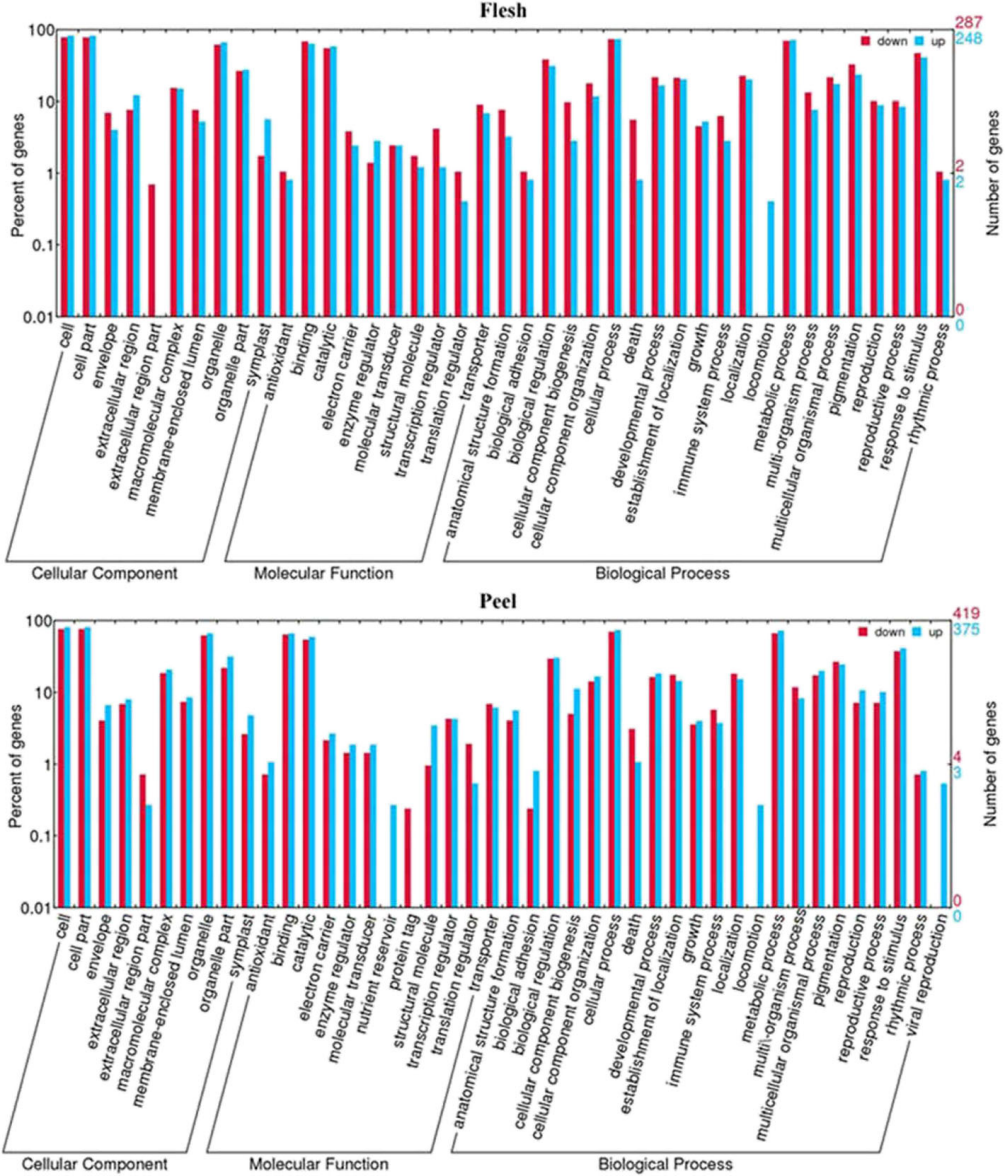
Go analysis of the DEGs up-regulated (blue) or down- regulated (red). The X-axis represents various GO terms in the three main categories of biological processes, cellular components, and molecular functions. The right Y-axis indicates the number of genes in a category. The left Y-axis indicates the percentage of a specific GO term of genes in the corresponding main category

A total of 20,944 genes were found to be present in at least one of the developmental stages in the flesh tissues of both WT and MT berries. Of these genes, 627 (2.99 %) had significantly differential expression (p ≤ 0.05) between WT and MT. Among these 627 differentially expressed genes (DEGs), 325 and 302 genes were up- and down- regulated in MT compared to WT, respectively. GO analysis revealed that these differential expression genes were involved in various different biological processes, molecular functions and cellular components in both flesh and peel tissue of MT (Fig. 6), suggesting that the mutation(s) in MT simultaneously up- and down- regulated different sets of genes involved in the same biological processes and functions. This observation was largely consistent with what was described for the uniquely expressed genes (Fig. 5) in which expression of some of the genes were detected only in MT or WT. Interestingly, the DEGs involved in the biological process- locomotion were all up-regulated in both flesh and peel tissues (Fig. 6) We also observed that some DEGs involved in the rhythmic process were either up- or down-regulated in both flesh and peel tissue, including PHYB (phytochrome b, VIT_205s0077g00940), GI (gigantea, VIT_218s0157g00020), CCR2 (glycine-rich rna-binding protein grp1a-like, VIT_203s0063g02610), CRY1 (cryptochrome 1 protein 2, VIT_218s0001g05680), PEX11C (peroxisomal membrane protein 11e, VIT_200s0120g00010) and some hypothetical protein (VIT_203s0038g02190, VIT_208s0105g00340). GO analysis of the 627 DEGs revealed that, within the term ‘Biological Process’, the most abundant categories were responses to stress, stimulus, reactive oxygen species and inorganic substance. KEGG enrichment analysis revealed only 5 pathways (out of 82 pathways covered) were significantly enriched (P ≤ 0.05). These pathways include vitamin B6 metabolism, terpenoid backbone biosynthesis, glycolysis /gluconeogenesis, peroxisome, and ascorbate and aldarate metabolism. When a FDR value 0.05 was used as the statistical threshold level, only 10 genes showed differential expression (Table 1). Among these 10 genes, F24J7.70 (tmv resistance protein n-like, VIT_200s0238g00060) and CSD1 (superoxide dismutase, VIT_214s0030g00950) were the two with largest and most significant differential expression between WT and MT (Table 1).

**Table 1:** Differentially expressed genes detected between WT and MT in the flesh and peel tissues (FDR < 0.05)

Seq. ID	Annotation	Gene name	GO term	log2Fold Change	*P* value	Padj	Tissue
VIT_200s0238g00060	tmv resistance protein n-like	F24J7.70	BP: defense response, signal transduction MF: ADP binding CC: intracellular	−4.275	3.71E-12	8.01E-08	Flesh
VIT_214s0030g00950	superoxide dismutase	CSD1	BP: oxidation-reduction process, response to ozone MF: superoxide dismutase activity, metal ion binding	−3.445	5.65E-09	6.09E-05	Flesh
VIT_212s0035g00410	disease resistance protein at3g14460-like	At3g14460	BP: defense response, signal transduction MF: ADP binding	−3.347	1.21E-05	0.0236	Flesh
VIT_205s0094g00350	class iv chitinase	EP3	BP: cell wall macromolecule catabolic process MF: chitinase activity CC: plant-type cell wall	−3.319	1.18E-07	0.0006	Flesh
VIT_210s0003g04505	hypothetical protein	--NA---	--NA---	−3.031	5.35E-06	0.0128	Flesh
VIT_207s0031g02630	aldehyde dehydrogenase family 3 member h1	ALDH3H1	BP: response to abscisic acid stimulus, oxidation-reduction process MF: aldehyde dehydrogenase [NAD(P)+] activity	−1.984	7.57E-08	0.0005	Flesh
VIT_213s0067g02270	uncharacterized protein	--NA---	BP: regulation of transcription, DNA-dependent, response to salicylic acid stimulus MF: transcription cofactor activity	−1.936	2.44E-06	0.0075	Flesh
VIT_219s0015g00320	calcium-activated outward-rectifying potassium channel 1	ATKCO1	BP: calcium ion transport MF: calcium-activated potassium channel activity	−1.903	2.20E-06	0.0075	Flesh
VIT_205s0049g00160	carnitine racemase like protein	IBR10	BP: fatty acid beta-oxidation, response to jasmonic acid stimulus, abscisic acid mediated signaling pathway CC: nucleus	−1.193	2.33E-05	0.0419	Flesh
VIT_201s0026g00430	---NA---	--NA---	BP: electron transport MF: electron carrier activity	3.652	7.65E-07	0.0033	Flesh
VIT_200s0238g00060	tmv resistance protein n-like	F24J7.70	BP: defense response, signal transduction MF: ADP binding CC: intracellular	−5.237	1.55E-20	2.79E-16	Peel
VIT_212s0028g02810	8-hydroxyquercetin 8-o-methyltransferase	OMT1	BP: methylation MF: O-methyltransferase activity	−4.229	3.76E-08	0.0001	Peel
VIT_215s0048g01680	cytochrome p450 monooxygenase cyp704g7	CYP704A2	BP: oxidation-reduction process, electron transport MF: oxidoreductase activity	−3.707	1.74E-06	0.0029	Peel
VIT_206s0004g07230	udp-glycosyltransferase 87-like	--NA---	BP: flavonoid biosynthetic process MF: indole-3-acetate beta-glucosyltransferase activity	−3.483	8.73E-06	0.0099	Peel
VIT_214s0030g00950	superoxide dismutase	CSD1	BP: oxidation-reduction process, response to ozone MF: superoxide dismutase activity, metal ion binding	−3.146	3.19E-10	1.44E-06	Peel
VIT_215s0046g02825	disease resistance protein rpp13	RPP8	BP: defense response MF: ADP binding	−3.105	4.18E-06	0.0058	Peel
VIT_215s0046g02750	disease resistance protein at1g50180-like	At1g50180	BP: defense response MF: ADP binding	−2.821	8.28E-06	0.0099	Peel
VIT_213s0067g01100	disease resistance protein at3g14460-like	At3g14460	BP: defense response MF: ADP binding	−2.770	5.12E-06	0.0066	Peel
VIT_214s0030g01150	unnamed protein product	--NA---	BP: oxidation-reduction process MF: superoxide dismutase activity	−2.476	1.88E-11	1.13E-07	Peel
VIT_213s0067g02270	uncharacterized protein	--NA---	BP: regulation of transcription, DNA-dependent, response to salicylic acid stimulus MF: transcription cofactor activity	−2.339	1.58E-13	1.43E-09	Peel
VIT_211s0016g04990	hypothetical protein	--NA---	--NA---	−2.304	2.30E-08	8.30E-05	Peel
VIT_219s0015g00320	calcium-activated outward-rectifying potassium channel 1	ATKCO1	BP: calcium ion transport MF: calcium-activated potassium channel activity	−1.804	4.04E-05	0.0347	Peel
VIT_205s0049g00160	carnitine racemase like protein	IBR10	BP: fatty acid beta-oxidation, response to jasmonic acid stimulus, abscisic acid mediated signaling pathway CC: nucleus	−1.401	1.23E-05	0.0131	Peel
VIT_209s0002g00550	zinc finger	GLIP1	BP: response to jasmonic acid stimulus, response to salicylic acid stimulus MF: lipase activity	−1.357	3.32E-05	0.0329	Peel
VIT_206s0080g00320	uncharacterized protein lo00248360	--NA---	MF: hydrolase activity CC: membrane	−1.351	6.19E-05	0.0466	Peel
VIT_202s0025g04620	phospholipase d	PLDBETA1	BP: response to cadmium io MF: phospholipase D activity CC: nucleus	−1.327	4.73E-07	0.001	Peel
VIT_218s0122g01290	rwp-rk domain-containing protein	RKD1	BP: regulation of transcription, DNA-dependent MF: protein binding	−1.114	3.64E-05	0.0329	Peel
VIT_200s0454g00010	hypothetical protein	--NA---	--NA---	1.011	4.85E-07	0.001	Peel
VIT_206s0004g05380	tropinone reductase homolog ag07440	SAG13	BP: oxidation-reduction process MF: oxidoreductase activity	1.436	3.01E-06	0.0045	Peel
VIT_206s0009g03640	annexin d8	ANNAT8	BP: response to salt stress MF: calcium ion binding CC: nucleus	1.824	5.23E-05	0.0411	Peel
VIT_209s0002g08465	pectinesterase qrt1	QRT1	BP: cell wall modification MF: aspartyl esterase activity	3.903	5.34E-07	0.001	Peel
VIT_211s0103g00110	photosystem ii protein d2	PsbD	BP: electron transport MF: iron ion binding	4.074	1.60E-07	0.0004	Peel

Of 20,804 genes whose expression were detected in the peels of both WT and MT, 905 genes (4.35 %) were differentially expressed (p ≤ 0.05). Among them, 794 genes were mapped to known genes with 375 being up-regulated and 419 genes down-regulated in MT compared to WT. These genes were involved in various basic functional categories, molecular processes and cellular components (Fig. 6). Genes responsible for biological processes of viral reproduction and locomotion and the molecular function of nutrient reservoir were all up-regulated (Fig. 6), while the genes for molecular function of protein tag was down-regulated. Interestingly, genes for the molecular process of locomotion was differentially expressed in both flesh and peel tissues. In the classification of molecular function, enrichment of GO terms is dominated by hydrolase activity acting on ester bonds, coenzyme binding, and oxidoreductase activity acting on paired donors and incorporating or reducing molecular oxygen. The statistically enriched pathways (corrected p-value < 0.05,) were glycolysis / gluconeogenesis, inositol phosphate metabolism, sesquiterpenoid and triterpenoid biosynthesis, ascorbate and aldarate metabolism, N-glycan biosynthesis, and peroxisome metabolic pathways.

When the FDR significant threshold at ≤ 0.05 was used, there were 22 genes which were differentially expressed in the peels between MT and WT (Table 1). They include up-regulated MT genes, such as PsbD (photosystem ii protein d2, VIT_211s0103g00110), QRT1 (pectinesterase qrt1, VIT_209s0002g08465), SAG13 (tropinone reductase homolog ag07440, VIT_206s0004g05380) and ANNAT8 (annexin d8, VIT_206s0009g03640), and down-regulated MT genes, such as GLIP1 (zinc finger, VIT_209s0002g00550), F24J7.70 (tmv resistance protein n-like, VIT_200s0238g00060), udp-glycosyltransferase 87-like (VIT_206s0004g07230), and OMT1 (8-hydroxyquercetin 8-o-methyltransferase,VIT_212s0028g02810). Among the DEGs (FDR ≤ 0.05) in peel and flesh tissues 5 were overlapped: F24J7.70, CSD1, ATKCO1 (calcium-activated outward-rectifying potassium channel 1, VIT_219s0015g00320), At3g14460 (disease resistance protein at3g14460-like,VIT_212s0035g00410, VIT_213s0067g01100) and IBR10 (carnitine racemase like protein, VIT_205s0049g00160).

### qRT-PCR validation of differentially expressed genes

**Fig. 7:**
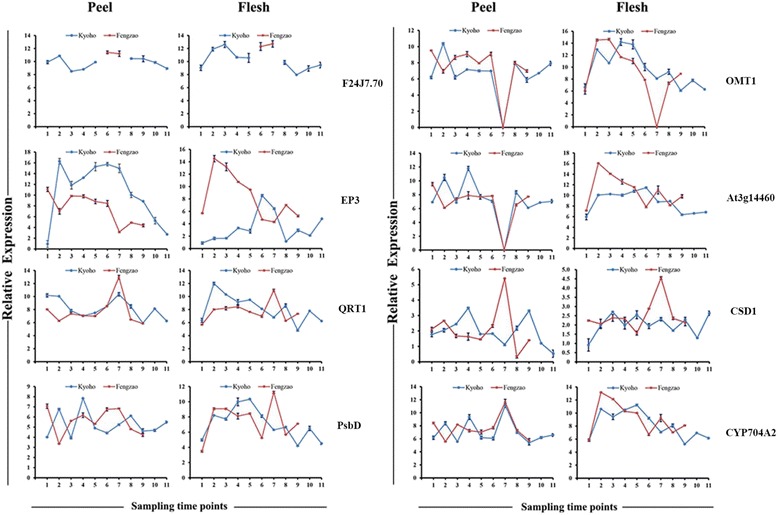
qRT-PCR expression patterns of 8 DEGs detected in the RNA-Seq profiles of WT and MT. The X-axis represents sampling time points corresponding to the 11 berry developmental stages of ‘Kyoho’ (see Fig. 1). The Y-axis represents the relative level of expression. The names of the assayed genes were indicated on the right sides of the panels: F24J7.70 (tmv resistance protein n-like), EP3 (class iv chitinase), QRT1 (pectinesterase qrt1), PsbD (photosystem ii protein d2), OMT1 (8-hydroxyquercetin 8-o-methyltransferase), At3g14460 (disease resistance protein at3g14460-like), CSD1(superoxide dismutase), and CYP704A2 (monooxygenase cyp704g7)

To assess the repeatability of the RNA-Seq sequencing data, we selected 8 genes of interest, which were detected highly differentially expressed between MT and WT in the RNA-Seq experiment, and determined their levels of qRT-PCR expression. These genes include F24J7.70 (tmv resistance protein n-like), EP3 (class iv chitinase), QRT1 (pectinesterase qrt1), PsbD (photosystem ii protein d2), OMT1 (8-hydroxyquercetin 8-o-methyltransferase), At3g14460 (disease resistance protein at3g14460-like), CSD1(superoxide dismutase) and CYP704A2 (monooxygenase cyp704g7) (Fig. 7, Additional file 1). The validation tests were carried out on the berry samples collected at the same berry developmental stages as that were used in the RNA-Seq experiment. The qPCR efficiency calibration curves for each of the 8 DEG primer pairs were established with the correlation coefficients ranging from 0.9827 to 0.9972) (Additional file 6). The qRT-PCR results from both peel and flesh tissues were fairly consistent with the respective RNA-Seq data and the mean Pearson correlation coefficient was 0.623. It was interesting to note that most of these 8 DEGs displayed lower transcript levels in MT than in WT (Fig. 7). The F24J7.70 gene (tmv resistance protein n-like gene) (Fig. 7, panel A) had distinct expression patterns between WT and MT. It only expressed around véraison (stages 6 and 7) in MT, but in all the berry developmental stages except the two stages around véraison in WT. On the other hand, the CSD1 (superoxide dismutase) gene displayed an expression peak in the period of véraison (stage 7) in MT but not in WT (Fig. 7, panel G).

## Discussion

K-means cluster analysis has been often used in characterizing and searching for gene expression patterns [22] and was conducted in this study. As expected, majority of the genes showed similar patterns of gene expression across different developmental stages between MT and WT. One interesting, but not unexpected, observation was that a subset of genes showed continuous increases in their expression during fruit development (cluster 9 in Fig. 4). These genes were presumably actively involved in berry ripening. Indeed, among them were several genes related to berry softening [23], including ripening-related protein grip22, class iv chitinase, l-ascorbate oxidase homolog and thaumatin-like protein. We also observed that many genes exhibited constant levels of expression across different developmental stages. As shown in Fig. 4 and Fig. 5, flesh and peel followed a parallel ripening trend, indicating that the same or very similar biological processes were involved in two different tissues during ripening.

There were quite numbers of genes which were only detected in WT (1127 in flesh and 950 in peel) or MT (1107 in flesh and 1228 in peel) at the experimental conditions and statistical analysis used in the this study. Lack of detectable expression for some of these genes in WT or MT was likely due to their relatively low levels of expression. Because most of these genes have not been annotated, their biological relevance to ripening could not be fully determined. Nevertheless, the facts that about 1,000 genes were uniquely expressed only in the flesh or peel tissue in either or both MT and WT and these genes were involved in many different biological processes and functions suggest that the early ripening mutation(s) in MT has pleiotropic impact on the expression of many genes and their associated networks. One interesting observation was the involvement of several genes related to the rhythmic process. Since these genes are known to be related to the signal transduction and flowering promotion, it would be interesting to investigate them further for determining how they might be involved in the early ripening in MT. A large number of QTLs related to grape phenological stages were recently identified, including those affecting berry ripening [24, 25]. QTLs for the onset of grape berry ripening (véraison) were mapped onto various linkage groups (LGs) by different research groups, including those on LGs 7 and 8 by Fischer et al. [26], LGs 2, 6 and 16 by Costantini et al. [25], LGs 2, 15 and 17 by Grzeskowiak et al. [27], LG 18 by Duchêne et al. [28], and LGs 17 and 18 by Mejía et al. [29]. Mean expression analysis in this study revealed several dozens of genes showing significantly differential expression between MT and WT and some of these genes were found in the general chromosomal regions of known QTLs reported. For example, CYP704A2 (cytochrome p450 monooxygenase cyp704g7, VIT_215s0048g01680, chr15:15882456.. 15885257) and VIT_215s0048g02610 (a hypothetical protein, chr15:16734823..16736027) showed significant differences in their expression levels between MT and WT and they were located in the same regions as the known QTLs/candidate genes related to the beginning stage of véraison (VIT_215s0048g00950, glutathione s-transferase zeta class-like glutathione s-transferase-like partial, chr15:15085137..15091168) and the end stage of véraison (VIT_215s0048g02000, homeobox-leucine zipper protein anthocyanin less 2-like, chr15:16132270..16139034) [27]. Similarly, a differentially expressed gene coding for a hypothetical protein identified in this study (VIT_211s0016g04990, chr11:4305018..4306207) was located in the region close to a reported QTL (chr11: 3,909,894 - 3,910,030, VVS2) related to véraison time determination on chromosome 11 [24].

Overall, the numbers of detectable DEGs between WT and MT were small for both flesh and peel tissue. When FDR signifcant threshold at ≤ 0.05 was used (fold change ≥1), there were only 10 and 22 genes which were differentially expressed in the flesh and peel tissues, respectively. Such a small number of detectable DEGs between WT and MT might suggest that the current experimental design was not sensitive enough to identify those DEGs which had smaller difference in the levels of their expression, but it might also suggest that the relative number of DEGs between WT and MT was indeed small. The latter possibility seems more likely, as the detecion of the mean expression difference between WT and MT for a gene was based on 8 different developmental stages (biological replicates) in this study. A comparion of the DEGs from the flesh and peel tissues revealed that 5 genes were commonly shared. Such high correlation (i.e. 5 genes shared between 10 and 22 DEGs observed in the flesh and peel tissue, respectively) cannot simply be explanied by coincidence. Instead, these shared genes might represent some common mechanisms contributing to the early berry ripening processs in both MT flesh and peel tissues.

One of the possible molecular mechanisms contributing to the early ripening of MT may be related to ROS. This hypothesis is supported by the revelation of a shared, commonly enriched GO term oxidoreductase activity from the enrichment analyses of the unique genes and DEGs and by the discovery of the CSD1 gene (superoxide dismutase,VIT_214s0030g00950) which was among the few genes most differentially expressed between WT and MT and shared in both MT peel and flesh tissues. There are extensive literature available in describing the important biological role of superoxide dismutases (SODs) as an important antioxidant defense against reactive oxygen species (ROS) damage [30] and a complex network of genes for fine tuning of ROS signals has been documented [31]. Fruit ripening, as an oxidative phenomenon, requires the removal of ROS such as H_2_O_2._ A balance between the production of ROS and their removal by antioxidant systems needs to be maintained [32]. Kumar et al. [33] recently determined the changes in ROS level during ripening of tomato wild type and *rin* mutant fruits, analyzed expression profiles of the corresponding genes in maintaining cellular redox state, and also found an important role of ROS during fruit ripening and senescence.

As a major class of antioxidant enzymes, SODs are important for removing biologically generated superoxide anion radicals. Their activities often change with the progression of the ripening processes in fruits. A decreasing trend in the SOD activity was observed during mango fruit ripening [34]. Similarly, Huang et al. [35] reported that the SOD activity decreased with maturation and ripening of sweet orange. Such decline in the SOD activity may be due to the accumulation of O^●−2^ and H_2_O_2_, which increased the oxidative stress during ripening. We observed in this study that the superoxide dismutase gene VIT_214s0030g00950 in MT and WT followed the similar patterns, but MT showed a much higher level of expression of the gene at the veraison stage and then reduced its expression more rapidly after veraison. qRT-PCR confirmed the RNA-Seq results that the expression of the superoxide dismutase gene was relatively low during initial berry development, increased gradually and reached the peak at véraison, and then decreased thereafter. The high-level expression of the superoxide dismutase gene at véraison was in agreement with the rapid accumulation of H_2_O_2_ at the stage as reported by others [36].

It is important to note that whether or not ROS acts as damaging, protective or signaling factors depends on the delicate equilibrium between ROS production and scavenging at the proper site and time. We observed in this study that the expression of SODs in MT decreased rapidly as berries approached ripening. This observation is consistent with other studies. Wu et al. [37] found that the expression of pectinesterase and Cu/Zn superoxide dismutase of ‘Fengjie 72-1’ was lower than that of its spontaneous late-ripening mutant. Ripening of tomato fruits was also accompanied by a progressive increase in oxidative/ peroxidative stress. The cultivar with short shelf life had higher oxidative stress than the cultivar with longer shelf life [38]. The reduced scavenging ability and associated increase in oxidative stress based on the reduction of SOD enzymes or genes may be responsible for mediating many of the physicochemical changes facilitating early ripening/softening of the fruits [38].

Possible involvement of ROS in ripening in this study was also supported by several other related gene activities. ROS accumulation can cause oxidative damage to mitochondrial proteins, resulting in dysfunction of various mitochondrial components, diseases and aging [39, 40]. One target of ROS signal transduction is the activation of Ca^2+^-permeable channels in plant membranes [31]. At the molecular level, the plant Ca^2+^-permeable Stelar K^+^ outward rectifier (SKOR) channel [41] and the Ca^2+^ conductance(s) involving annexin1 [42] have been shown to be responsive to H_2_O_2_. A model that could integrate the ROS and Ca^2+^ with electric signal waves was outlined by Gilroy et al. [31]. H_2_O_2_ promotes a positive feedback mechanism on active SKOR channels [43]. Interestingly in this study, we observed that the expression of ATKCO1gene (calcium-activated outward-rectifying potassium channel, VIT_219s0015g00320) was significantly reduced in both peel and flesh tissues of MT (Table 1), possibly as a result of reduced ROS activities. Another support evidence is that we observed ANNAT8gene (annexin d8, VIT_206s0009g03640) in the peel of WT was up-regulated. Previous studies have indicated that plant annexins have key roles in the cross-talk between calcium and reactive oxygen species (ROS) under stress signaling [44]. In *Arabidopsis* annexin 1 was found to mediate a plasma membrane calcium-permeable conductance in roots that is activated by reactive oxygen species [42].

The decrease of SOD gene expression in MT might suggest that the oxidative stress was higher in MT than in WT. An increase in the production and accumulation of reactive oxygen species (ROS) can result in an increase in membrane lipid peroxidation, thus injuring the integrity of cellular structure [40]. This type of changes often results in extensive cell wall degradation which reduces fruit firmness during the process of ripening. Previous reports describing the dynamic expression profiles of cell wall-modifying enzymes and corresponding changes in cell wall composition during berry ripening have shown that modification of pectins was primarily responsible for the progressive loss of firmness in ripening fruits [45, 46]. It was reported that pectinesterase removed methyl groups of the wall galacturonans to enhance depolymerization by both endo- and exopolygalacturonase [47]. Pectinesterase (PE) (EC 3.1.1.11) is a ubiquitous cell-wall-associated enzyme which facilitates plant cell wall modification and subsequent breakdown. The inability to form calcium cross-bridges would lead to cell separation and affect fruit integrity [48]. Seventy-five percent of berry pectin is located in the skin [5]. In this study, we found that the expression of QRT1 (VIT_209s0002g08465, pectinesterase qrt1), which encodes pectinesterase qrt1 involved in the process of pectin dedragration, was much higher in MT than WT (Table 1). This observation, along with several lines of evidence described earlier, led us to hypothesize that the ROS level in MT was higher than that of WT at veraison and maintained below the damaging level, but high enough to accelerate the ripening process in MT. It was interesting to note that we did not find any DEGs related to plant hormones which are well-known major regulators involved in the fruit ripening processes of many species, including grapes. This could be due to the detection limit of DEGs under the current experimental condition and design. An alternative explanation is that some or any of the potential ABA effect might be offset by the ROS effect we observed, since ROS has been reported to be able to enhance ABA biosynthesis or inhibit ABA degradation [49].

One other possible ripening mechanism discovered in this study involves pathogenesis-related (PR) proteins. While PR proteins are generally considered as plant defense proteins, several previous studies showed that the induction and expression of PR-genes with and during fruit ripening were not related to induction by pathogens but developmentally controlled and induced via grape fruit ripening signals [4, 50]. Other reports also suggested that PR proteins were synthesized in healthy grape berries in a developmentally dependent manner as a normal part of the ripening process [8, 51]. We observed several differentially expressed genes which are related to disease resistance proteins (Table 1). The At3g14460 gene (disease resistance protein at3g14460-like, VIT_213s0067g01100) was among the most differentially expressed genes detected in both peel and flesh tissues. F24J7.70 (TMV resistance protein n-like, VIT_200s0238g00060) was another PR-related gene among the most differentially expressed genes detected in this study. The levels of expression of TMV resistance protein n-like varied considerably at different developmental stages between WT and MT (Fig. 7). Interestingly, Pirona et al. [52] annotated a gene of TMV resistance protein N when they analyzed the *qMD4.1* locus which was a QTL controlling maturity date in peach. Lijavetzky et al. [3] also observed the activation of pathogen defense gene expression responses in the pericarp upon berry ripeness in ‘Muscat Hamburg’.

Accumulation of PR proteins has also been demonstrated during ripening of other fruits [8, 53, 54]. For example, large-scale analysis of gene expression differences in peels between late ripening cultivar of citrus and its wild type revealed that 7 genes were significantly differentially expressed, including two disease resistance-responsive protein-related genes and one chitinase gene [55]. The two disease resistance-responsive protein-related genes and chitinase gene were all up-regulated in the late ripening cultivar. Another example is that two defense-related proteins, pathogenesis-related protein STH-2 and like, were found to accumulate at significantly higher levels in Green-ripe (*Gr*) tomato mutant fruit than that in the wild type [56]. It is interesting to note that the expression of PR genes and chitinases gene in the early ripening “Fengzao” mutant in this study were down-regulated, which was in line with the expectation from citrus and tomato studies above. Chitinases (E.C. 3.2.1.14) are glycosyl hydrolases which were implicated in the protection of plant tissues against fungal pathogens [57] due to their ability to hydrolyze chitin, a component of fungal cell walls. However, these proteins may have a role in normal growth and development as well [58]. Indeed, proteomic studies during grape berry ripening noted a significant increase in chitinase and 1,3-_β_-glucanase activity and antigen levels between harvest and the fruit becoming ripe [59].

In conclusion, fruit ripening is a well-regulated process in which many genes are likely involved. Disruption or alteration of the function of a critical gene(s) in the process can result in changes of expression profiles of many related genes. While the nature of the mutation responsible for early ripening in ‘Fengzao’ is unknown, comparative profiling of the berry development between ‘Fengzao’ and its wild type ‘Kyoho’ suggested that ROS and PR related genes might play critical roles. Some genes responsible for cell wall degradation and plasma membrane might be involved as well.

## Additional files

Additional file 1: Primer sequences used in the qRT-PCR experiment. (XLS 26 kb)
Additional file 2: Alignment and mapping results of the Illumina RNA-Seq reads in this study. (XLSX 11 kb)
Additional file 3: Expression clusters, annotation, and functional categories and distribution of 20,944 genes expressed in the flesh tissues of both WT and MT across various developmental stages. (XLSX 355 kb)
Additional file 4: Expression clusters, annotation, and functional categories and distribution of 20,804 genes expressed in the peel tissues of both WT and MT across various developmental stages. (XLSX 359 kb)
Additional file 5: GO analysis results of the genes expressed only in the flesh or peel tissue of WT or MT. (XLSX 69 kb)
Additional file 6: qPCR efficiency calibration curves for each of the 8 pairs of primers used in the study. The X-axis represents Ct and the Y-axis represents log_10_ DNA concentration. The names of the assayed genes were indicated on the right side of the panels. The genomic DNA of ‘Fengzao’ was serially diluted twice to a constant content of 10 ng of genomic DNA per ul. Each dilution was measured twice. (PDF 84 kb)
