# Supplementary figures and images for: Comparative RNA-Seq profiling of berry development between table grape ‘Kyoho’ and its early-ripening mutant ’Fengzao’
Source: BMC Genomics. 2016 Oct 12;17:795. doi: 10.1186/s12864-016-3051-1 (PMC5059895; doi:10.1186/s12864-016-3051-1)

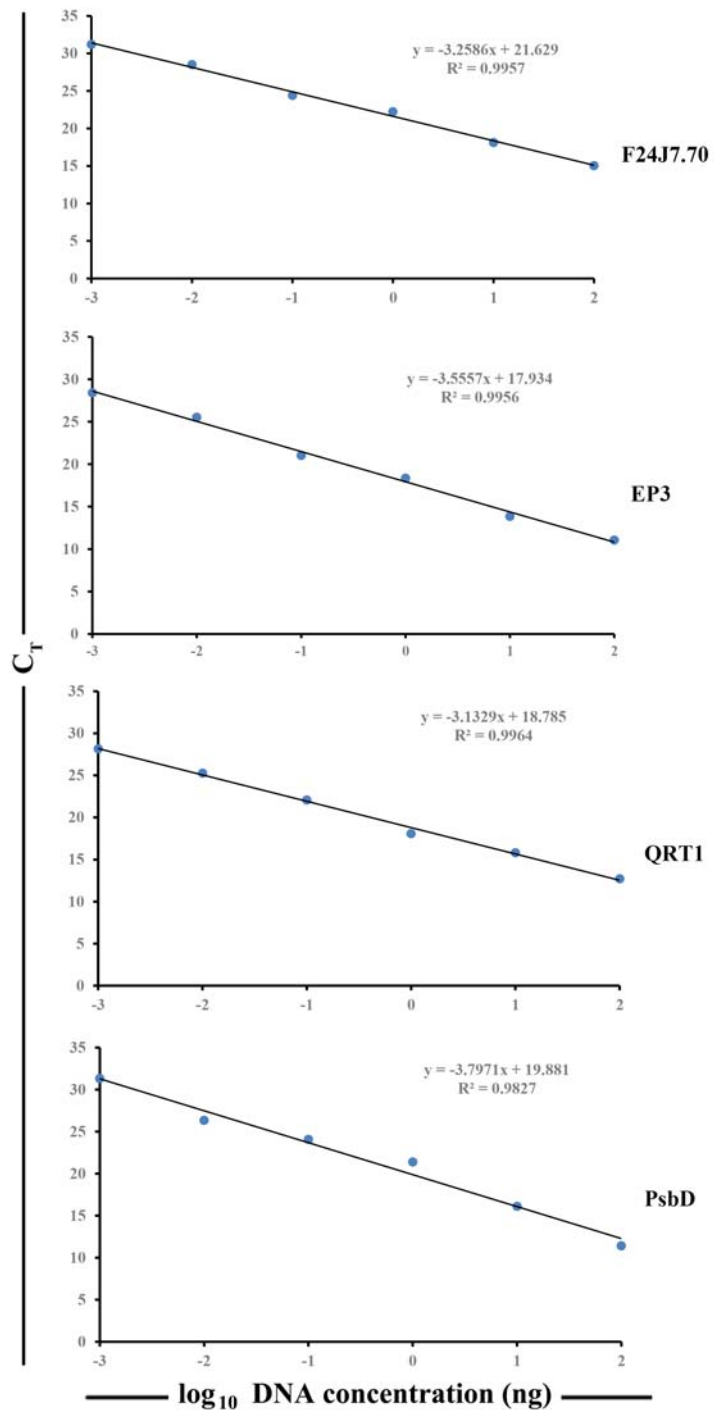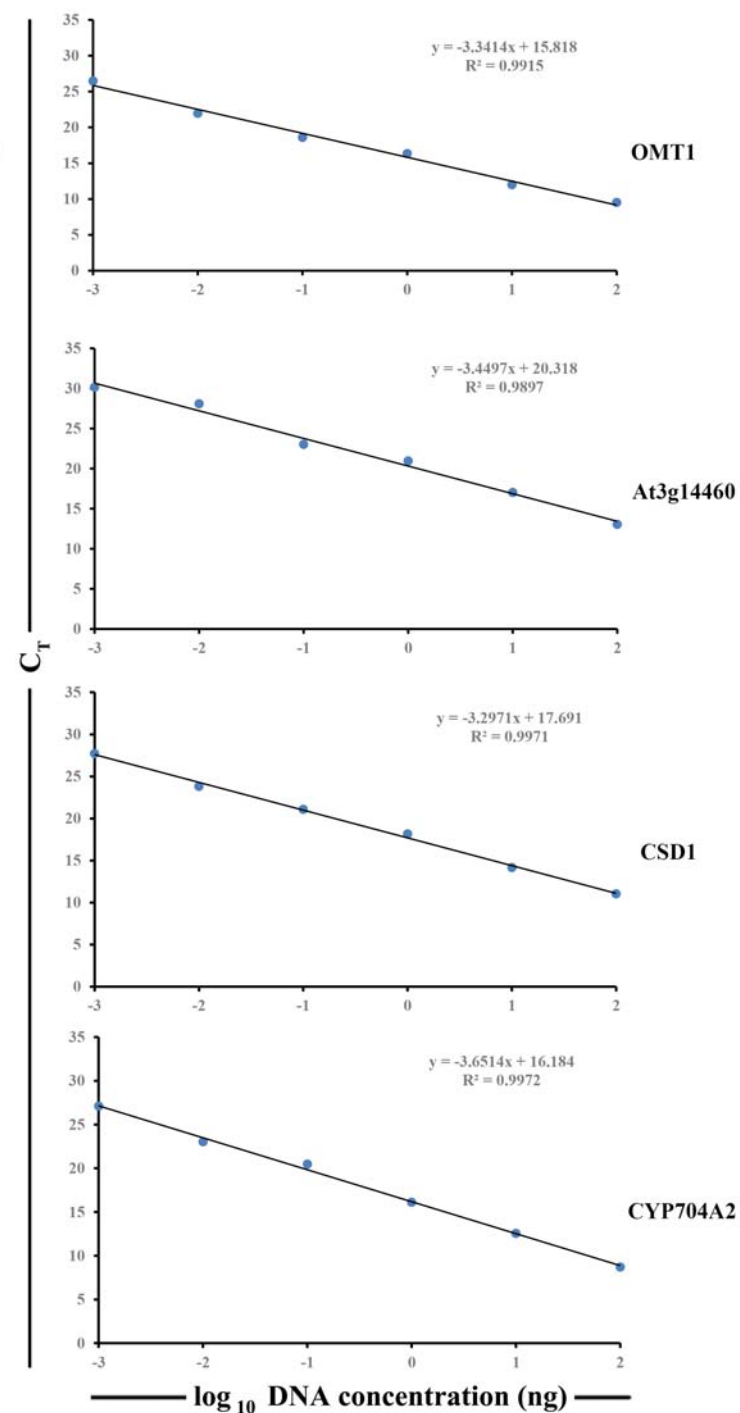

Supplement: Additional file 6: — qPCR efficiency calibration curves for each of the 8 pairs of primers used in the study. The X-axis represents Ct and the Y-axis represents log10 DNA concentration. The names of the assayed genes were indicated on the right side of the panels. The genomic DNA of ‘Fengzao’ was serially diluted twice to a constant content of 10 ng of genomic DNA per ul. Each dilution was measured twice. (PDF 84 kb) [file 12864_2016_3051_MOESM6_ESM.pdf]
